# Development of easy-to-notice nurse call with low stress: a pilot study using heart rate variability

**DOI:** 10.1539/eohp.2025-0010

**Published:** 2026-04-16

**Authors:** Mako Katagiri, Masao Ohira, Yoshiaki Sakurai

**Affiliations:** 1 Research Division of Product Reliability, Osaka Research Institute of Industrial Science and Technology, Osaka, Japan; 2 President’s Office, CARECOM Co., LTD, Tokyo, Japan; 3 Equipment Sharing Center for Advanced Research and Innovation, Osaka Metropolitan University, Osaka, Japan

**Keywords:** cardiac sympathetic index, heart rate variability, nurse call, RR-interval, stress, tremolo

## Abstract

**Objectives:**

This study evaluated sounds used in nurse call (NC) systems to reduce stress in the nursing work environment by developing low-stress NCs.

**Methods:**

Participants’ noticeability and stress were evaluated based on their heart rate variability while listening to nine types of test sounds, including two existing NCs. Next, a new NC was extracted from the test sounds and was verified in the field as a replacement for the tremolo sound (tremolo), which has a negative impression.

**Results:**

Tremolo was the most noticeable. However, this sound generates the highest cardiac sympathetic index (CSI), which is an index of stress-related sympathetic nerve activity. Therefore, we extracted a C sound (C6 + G#6), which is the second most noticeable sound after tremolo. The new sound was introduced into a NC system, and the CSI of nurses on duty was analyzed. We noticed that the C sound was less stressful than tremolo.

**Conclusions:**

The findings suggest that an improvement in the NC sound quality may lead to reduced stress among nurses in the work field.

## Introduction

We experience stress in our daily lives. Although stress is a motivating emotion, it can adversely affect the human body when experienced for long periods. Stress is characterized by physiological changes, such as increased blood pressure, increased pulse rate, and decreased alpha wave activity in the brainwaves^1)^. For nurses, whose job is to save lives, stress is an unavoidable problem. Various stressors during work are closely related to the work environment, including nurse call (NCs). Various studies have investigated the stress faced during work using heart rate variability^2,3,4,5)^. However, to our knowledge, no previous studies have investigated the effects of NCs on stress assessed using heart rate variability (HRV) data.

NCs are said to originate from the “doorbell” invented by Florence Nightingale^6)^. Nevertheless, NCs do not have a specific international standard. Instead, individual standards have been developed, such as the UL1069 NC system in the United States and the DIN VDE 0834 call system in Germany. In Japan, Industrial Standards^7)^ and the Intercom Industry Association established voluntary standards^8)^. NCs consist of several types of sounds installed in NC systems in hospitals and long-term care insurance facilities. Patients use these systems to call nurses, caregivers, and other medical staff members. Therefore, NCs must accurately detect and relay subtle sounds to ensure effective communication between patients and medical staff. However, NCs may not be noticed or heard during the day because of ambient noise or under certain conditions at night. In addition, NC systems are connected to various medical devices and are responsible for ringing alarms from these devices. Consequently, the number of alarms issued each day is increasing, and NC itself is a noise source, stressing the medical staff.

A large-scale survey report^9)^ covering approximately 1,000 nurses from nine hospitals nationwide reported that most nurses felt that the current NC sounds, especially the Tremolo used for emergency calls and bathroom/toilet call sounds, were unpleasant and needed to be changed. Although previous studies on nurse stress have focused on the effect of the device alarm^10)^ and alarm development^11,12)^, no studies have investigated the relationship between heart rate and NCs with respect to nurse stress.

To ensure that the alarm can be clearly heard, the sound pressure level of an auditory alarm in the workplace or public space should be at least 15 dB higher than the sound pressure level of background noise, and the frequency band should be set between 500–4,000 Hz, where human hearing is sensitive^13)^. Moreover, increasing the sound pressure level increases the perception of sound as an auditory signal. However, an increased sound pressure level can also increase stress at staff stations, where various sounds are generated (a previous survey^9)^ noticed that staff stations are overwhelmingly noisy).

This study aimed to understand the subjective impressions of the nurses’ susceptibility to NCs and stress based on heart rate variability. We also aimed to develop a new NC that is less stressful without increasing the sound pressure level.

## Methods

### Sound environment survey in staff station

**Fig. 1.**
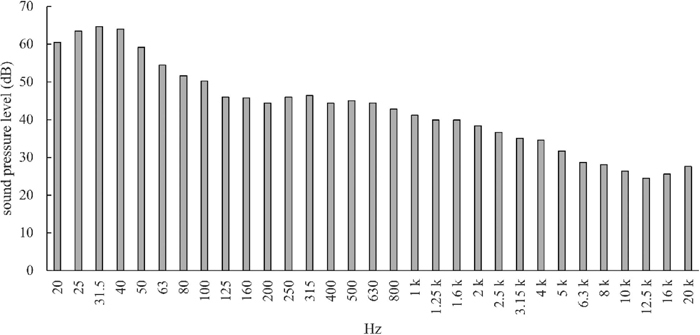
Frequency analysis results of sound pressure level at 12:40 in 3A staff station.

To survey the sound environment in the work field, a precision sound level meter (NA-28: RION Co., Ltd., Tokyo, Japan) was installed at the staff station of Ward 3A, Sapporo Kosei Hospital of JA Hokkaido Koseiren (Hokkaido Federation of Agricultural Cooperatives), and the sound pressure level was measured from 08:30 to 16:30 on weekdays. Fluctuations in the sound pressure levels were measured every 5 min and recorded on a dedicated memory card. The average sound pressure level on the measurement day was 70.2 dB, with slight variation. Figure 1 shows the frequency analysis results at 12:40, when the sound pressure level was the highest at 72.2 dB (A-weighted sound pressure level was 51.7 dBA). The sound environment in the staff station of Ward 3A was characterized by relatively higher energy at lower frequencies, peaking at 31.5 Hz.

### Listening experiment in a conference room assuming off-duty hours

#### Preparation for test sound

The audible frequency range for humans is 20 Hz–20 kHz^14)^, of which 2–5 kHz is considered a band with high auditory sensitivity^15)^. In addition, the Japanese Industrial Standard specifies that alarm tones should not exceed 2.5 kHz^16)^. Thus, seven chords of different pitches that correspond to the low frequency band of 125–160 Hz and the frequency band above 800 Hz were selected based on the Measurement and Analysis Methods of Human Physiological Responses^17)^. Nine test sounds were considered in the experiment, including two types of NC (chime and tremolo) in NC systems (BZM-002WZ) manufactured by CARECOM Inc (Tokyo, Japan). The test sounds used in this experiment were created using acoustic processing software (Pro Tools; Avid Technology, Boston, MA, USA). Moreover, preventive measures were implemented to avoid clicking sounds during playback. Each test sound lasted 1 s and was played twice, with 1 s of silence. The presentation interval was 15 ± 3 s, and the sounds were randomly presented twice. Table 1 lists the sounds used in these experiments. All sound outputs were adjusted to the same level.

**Table 1.** Nine test sounds used in the experiment including tremolo and chime

**Test sound**	**Pitch Name**	**Frequency (Hz)**
Chime	―	1319 + 1568
Tremolo	―	2088 + 2640
A	C3E3	130.8 + 164.8
B	C5G#5	523.3 + 830.7
C	C6G#6	1046.5 + 1661.4
D	C6E6	1046.5 + 1318.6
E	A5A6	440 + 880
F	A6A7	880 + 1760
G	C6C7	1046.5 + 2093

#### Participants

Ten nurses working at a hospital, aged 22–37 (mean 30.1; standard deviation [SD], 5.7) years, participated in this study. Although hearing tests were not performed, they were deemed to have generally normal hearing, as they had no medical history of hearing problems. Before each experiment, the participants were briefed on the purpose of the study and provided written informed consent. This study was approved (IZH 2602) by the Steering Committee (Ethics Committee) of the Osaka Research Institute of Industrial Science and Technology and was conducted in accordance with the Declaration of Helsinki. The profiles of the ten participants are as follows: two males and eight females, four with less than 3 years of work experience (including both males), two with 4–10 years of experience, and four with 11 years or more.

#### Procedures and methods

Two types of listening experiments were consecutively conducted in a conference room in the hospital, where the participants were assumed to be off duty. In the first experiment, 10 participants were exposed to a 20-second set of ringing chime and a tremolo, both commonly used in hospital wards, and their stress levels were compared when exposed to each sound. Subsequently, the noticeability and stress with respect to the nine test sounds were investigated. All data were evaluated based on participants’ heart rate variability. The test sounds were output from two speakers (LSPX-103E26, Sony Corporation, Tokyo, Japan) installed in the room, and these volumes were adjusted to be equivalent to the background sound of 62.0 dB in the conference room. The distance between the left and right speakers and the participants was 2.5 m, and they were arranged to form an equilateral triangle. In addition, before the experiment, it was confirmed that no differences were observed in the test sound level depending on the seating position. During the experiment, participants were instructed to talk, read, eat, and drink freely and without focusing on the sounds. The experiment was conducted with a maximum of four participants for simultaneous measurements. The duration of the experiment was approximately 30 min.

#### Measurements

To prevent noise contamination during the measurement of heart rate variability, the participants wiped their skin with alcohol to lower the skin contact impedance, and portable electrodes were affixed to three locations near the participant’s left belly (anode), right clavicle (cathode), and left subclavicle (ground) based on the three-point induction method. The participants carried a small electrocardiogram (ECG) amplifier (PolyTele; NIHON SANTEKU Co., Ltd., Osaka, Japan) to measure their heart rate. The measured data were stored on a computer using autonomic nerve measurement software (MaP1058; NIHON SANTEKU Co., Ltd.). The sampling frequency for obtaining the electrocardiogram was 1000 Hz.

#### Physiological assessment for test sounds

The measured ECG waveforms were visually checked for artifact contamination, and the peak of the R wave was detected and evaluated based on the time interval between the detected and adjacent R waves (RR interval [RRI]). The stress for the test sounds was determined using the cardiac sympathetic index (CSI) of autonomic nervous system activity derived from the Lorenz plot analysis based on the RRI^18)^. The CSI values were derived from an autonomic nerve analysis program (MaP1060; NIHON SANTEKU Co., Ltd). The noticeability of the test sounds is based on a physiological phenomenon called the Orienting Response (OR), in which the RRI changes when an organism unexpectedly becomes aware of an external stimulus^19)^. Specifically, the RRI for the five beats before the sound presentation was called RR1 to RR5, and the RRI for the five beats after the sound was called RRI6 to RRI10. The average (M1 and M2) of the five beats before and after the sound presentation was calculated as follows:

Before

$$M1=\frac{\left(RR1+RR2+RR3+RR4+RR5\right)}{5}$$

$$SD=\frac{\sqrt{{\left(RR1-M1\right)}^{2}+{\left(RR2-M1\right)}^{2}+{\left(RR3-M1\right)}^{2}+{\left(RR4-M1\right)}^{2}+{\left(RR5-M1\right)}^{2}}}{5}$$

After

$$M2=\frac{\left(RR6+RR7+RR8+RR9+RR10\right)}{5}$$

OR-Value: $\frac{\left|M1-M2\right|}{SD}≧2$

Moreover, the sound was judged as noticeable when the OR value changed by ±2 SD or more^20)^. The OR values obtained from repeating each test sound twice were converted into categorical data ranging from 0 to 2 (0: not noticed both times, 1: noticed once, 2: noticed both times). In addition, the frequency of easy-to-notice sounds in terms of percentage (%) for each test sound was calculated by dividing the number of valid OR-value times by the number of presentations (ie, 2).

#### Statistical methods

Paired t-tests were used to compare chimes and tremolos. McNemar’s test was used to compare tremolos with alternative sounds selected from the test sounds. All analyses were performed using predictive analysis software (JMP17.0; SAS Institute Inc., Cary, NC, USA) and statistical significance was set at *p*<0.05 (two-tailed test).

## Results

**Fig. 2.**
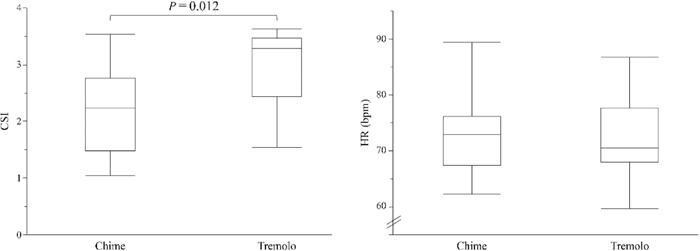
Comparison of CSI and HR when hearing chime and tremolo. Each bar represents the mean value of (n=10), and error bars indicate the standard deviation. A statistically significant difference in average CSI was observed (*p*=0.012). CSI, cardiac sympathetic index; HR, heart rate.

Figure 2 shows the average heart rate and CSI results of the ten participants. No significant difference was found in the average heart rate for each sound exposure for 20 s (t(9)=−0.796, *p*=0.498). The mean difference was −0.80 (95% confidence interval [CI], −3.35 to 1.75). However, a significant difference was observed in the average CSI, a stress index (t(9)=3.115, *p*=0.012). The mean difference was 0.77 (95% CI, 0.21–1.32), and the post-hoc power for this sample size was 52%.

**Fig. 3.**
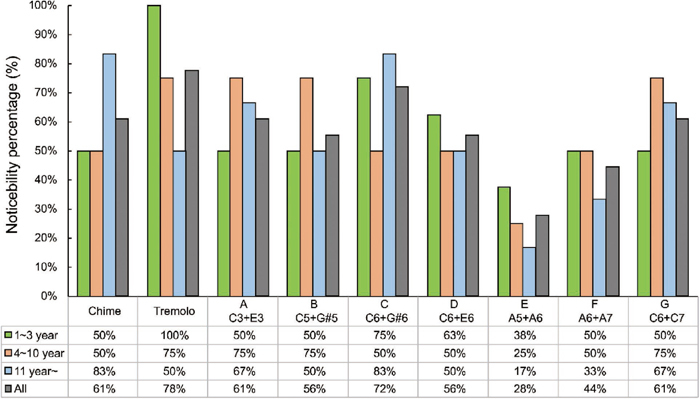
Comparison of the noticeability percentage of test sounds by nursing career.

Figure 3 shows a comparison of the noticeability percentages of the nine test sounds according to nursing experience. Of the ten participants, one with over 11 years of experience was excluded from the analysis because the ECG waveform presented several artifacts. Among the test sounds, tremolo was the easiest to notice, with 78% noticeability. In particular, participants with less than 3 years of nursing experience recognized tremolo 100%, indicating that they were quite sensitive to tremolo. The results of a non-parametric test (Kruskal-Wallis test) showed that tremolo had a statistically significant difference in nursing career and noticeability (*p*=0.045). Moreover, of the seven test sounds produced in this study, the noticeability of the C sound (C6+G#6) was 72%, which was comparable to that of the tremolo. McNamar’s test was used to evaluate the difference between tremolo and C sound noticeability; no significant difference was observed (*p*=0.655).

### Verification in the working ward

#### Preparation of C sound

**Fig. 4.**
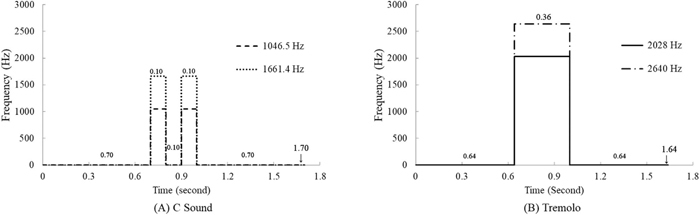
C Sound and tremolo frequency values for the sound duration. (**A**) The constituent frequencies of C-sound are 1046.5 Hz and 1661.4 Hz. (**B**) The constituent frequencies of tremolo are 2028 Hz and 2640 Hz.

The pattern of the C sound was created, as shown in Figure 4, considering that the on/off pattern of tremolo was at 1 s intervals.

#### Participant

A male nurse with less than 3 years of nursing experience participated in a conference room experiment.

#### Procedures and methods

Two experiments were conducted in the work ward, initially with a conventional tremolo-equipped NC system and 10 months later with a C-sound-equipped NC system. Before starting normal work, the participant was fitted with disposable electrodes and a small polygraph telemeter similar to that used in the conference room experiment. The measurement time was approximately 8 h. In addition, a video camera was installed at the staff station to confirm whether a nurse wearing an electrocardiogram was in the staff station when the tremolo or C sounds were played.

#### Comparison of tremolo and C sound

**Fig. 5.**
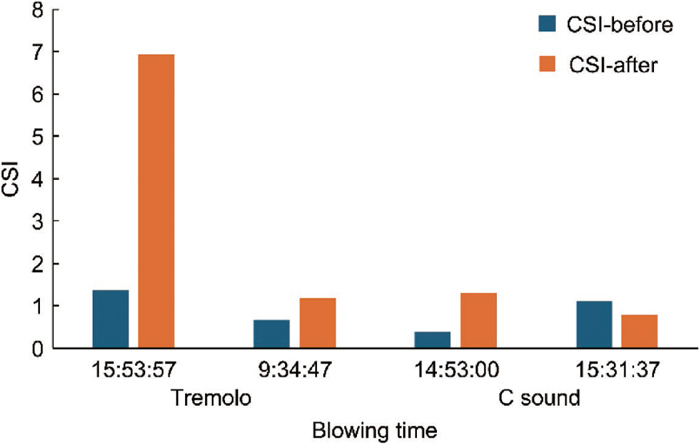
Comparison of CSI before and after activating tremolo and C sounds. CSI, cardiac sympathetic index.

For the system with tremolo, the total number of NCs activated at the staff station during the working hours of the participant was 11. The tremolos were activated twice (at 10:40:08 and 15:53:57). In contrast, 34 NCs were counted using the C sound system, of which eight were C sounds. The heart rate variability data that could be measured because the participant was at the staff station were one tremolo (15:53:57) and three C sounds (9:34:47, 14:53:00, and 15:31:37). Figure 5 compares the changes in the CSI before and after ringing the tremolo and C sounds. For tremolo, the CSI increased rapidly after activation, whereas the C sound showed a smaller increase in CSI after activation.

## Discussion

Based on a survey of nurses’ attitudes toward NC, we focused on tremolo, which nurses found unpleasant and wanted to change. We then analyzed the relationship between the frequency of awareness and stress and the changes in heart rate variability. Tremolo was the easiest to notice, and this tendency was more pronounced in the early stages of nursing careers. When compared with the existing chime, the CSI was significantly higher. Based on the available data, we observed that tremolo NC increased the participant’s stress. In other words, although tremolo is highly noticeable, it causes stress. In particular, nurses are sensitive to tremolo because this sound is often used to call for emergencies, toilets, and baths.

Furthermore, to consider alternative sounds to tremolo, we created seven types of sounds with different frequencies, considering the sound environment of the nursing station. The tested sounds, including the two existing types of NC, were presented randomly twice, and the heart rate fluctuations of the participants in response to the sounds were measured. Among the test sounds, we selected the C sound, which was the second most noticeable sound after tremolo. Subsequently, we included this sound in the NC system to compare the stress levels between the conventional tremolo and the C sound. In the work ward, data were obtained once and three times for the tremolo and C sounds, respectively, and the easy-to-notice levels by OR values were 10.34 for the tremolo, and 2.38, 2.45, and 0.53 for the C sound. The third C sound at 15:31:37 had a noticeability of 2 or less, and there was almost no change in the CSI. Thus, the participants might not have noticed the C sound. In contrast, after tremolo calling, which had a noticeability of two or more, the CSI increased, and a similar trend was observed for the C sound.

The results indicated that the participants were alerted or tense in response to the sounds. However, the increase in CSI for the C sound was significantly lower than that of tremolo. In other words, the results suggest that even if the C sound is activated at an unexpected time, its effect on the participants is small. Therefore, the C sound may be a suitable substitute for tremolo as an NC that is easy-to-notice and less stressful. Currently, Sapporo Kosei Hospital is using this C sound instead of the tremolo. Thus far, the novel sound has been well-received.

A limitation of this study is that tremolo and C sound were compared in one participant. In addition, because the measurements were obtained during working hours, the experimental data could not be obtained when the participant was not at the staff station, even when the target sound was ringing. Therefore, it cannot be said that the novelty of the C sound or habituation of the C sound through continued use was considered. Furthermore, the relationship between nursing experience and changes in NC discomfort has not been fully examined. The reason for the decrease in discomfort with the C sound compared to tremelo, whether it is due to habituation or calmness, and the relationship between subjective evaluations and physiological responses must be studied. Regarding the C sound currently in use, we plan to conduct a follow-up survey with a larger sample size, with the cooperation of the hospital. We aim to support new efforts to improve the working environment of nurses and the living environment of patients.

Potential alarm-related problems in the nursing field have also been investigated. For example, studies on alarm fatigue emitted from medical devices, including NCs^21)^, and evaluations of NCs from the aspect of nursing management have been reported^22,23)^. Some studies have highlighted the importance of new alarm systems^24,25)^. The results of this study suggest that the developed low-stress NC can reduce the stress of medical personnel and improve the safety and quality of patient care. Therefore, we suggest that this novel sound can be applied in clinical settings to improve the nursing environment.

## Conclusion

Using heart rate variability, we investigated the NCs in terms of their noticeability and degree of stress. We found that the existing tremolo was noticeable as a NC but was stressful. Therefore, to develop a noticeable and stress-free sound, test sounds with different frequencies were created, considering the sound environment of a hospital ward. Moreover, we extracted a C sound (C6 + G#6) that has the same level of noticeability as tremolo from the heart rate variability analysis of the participants when exposed to the test sound. We compared the noticeability and stress caused by sounds in the work ward using NC systems equipped with tremolo and C sounds, respectively. The results indicated that the increase in CSI when the sound was noticed was smaller for the C sound than for the tremolo sound, which produced a sudden increase. Consequently, we conclude that the C sound was easier to notice and less stressful. We proposed the C sound as an alternative to tremolo. This sound is currently being introduced into the NC ward system of a cooperative hospital. Thus far, the novel sound has been well-received and is being used.

## Data Availability

The datasets used and/or analyzed during the current study are available from the corresponding author upon reasonable request.
